# Supplementary material for: A New Plasmacytoid Dendritic Cell-Based Vaccine in Combination with Anti-PD-1 Expands the Tumor-Specific CD8+ T Cells of Lung Cancer Patients
Source: Int J Mol Sci. 2023 Jan 18;24(3):1897. doi: 10.3390/ijms24031897 (PMC9915756; doi:10.3390/ijms24031897)
Supplement: Supplementary file 1 [file ijms-24-01897-s001.zip › ijms-2101562-supplementary.pdf]

Supplementary Materials

A New Plasmacytoid Dendritic Cell-Based Vaccine in Combination with Anti-PD1 Expands the Tumor-Specific CD8+ T Cells of Lung Cancer Patients

Dalil Hannani[1], Estelle Leplus[1], David Laurin[2,3], Benjamin Caulier[2,3], Caroline Aspord[2,3], Natacha Madelon[2,3], Ekaterina Bourova-Flin[4], Christian Brambilla[3,5], Elisabeth Brambilla[3,5], Anne-Claire Toffart[3,5], Karine Laulagnier[1], Laurence Chaperot[2,3], Joël Plumas[1,2,*]

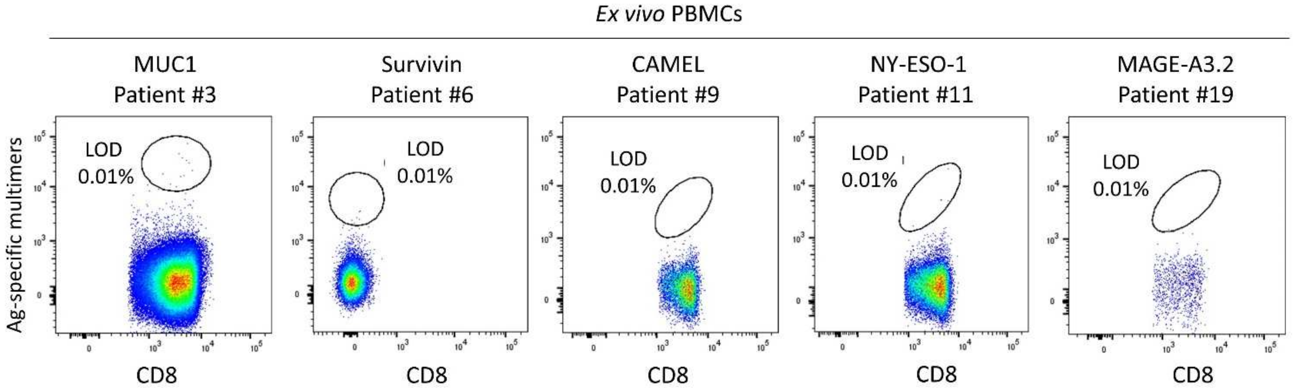

Figure S1. Baseline frequency of tumor antigen-specific T cells in lung cancer patients' PBMCs. Patients' PBMCs were labeled with multimers specific to the indicated antigens as described in Materials and Methods. Results for five patients and five antigens are shown and correspond to the same patients and antigens illustrated in Figure 2. In none of the examples, the baseline frequency exceeds the limit of detection (LOD) of the assay, i.e., 0.01% of total CD8+ T cells.

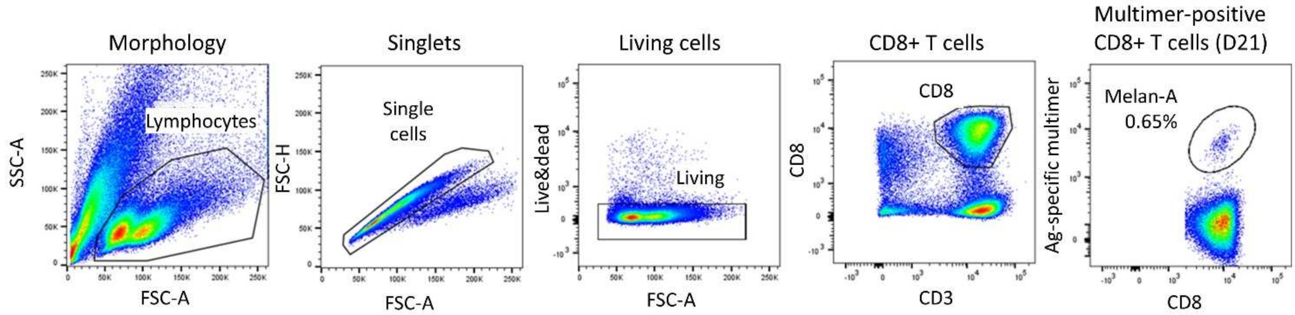

Figure S2: Gating strategy for the study. Lymphocytes were selected based on their morphology in SSC versus FSC dot plots. Then, singlets living CD8-positive cells were successively selected before gating the population of multimer-positive cells to determine the frequency of tumor antigen-specific CD8+ T cells. The example on the right shows the proportion of Melan-A-specific CD8+ T cells detected after coculture of patients' PBMCs with Melan-A-loaded PDC*line cells.
